# Potential Therapeutic Effect of Citronellal on Diabetic Cardiomyopathy in Experimental Rats

**DOI:** 10.1155/2021/9987531

**Published:** 2021-11-17

**Authors:** Jun-Xiu Lu, Yue Qiu, Li-Juan Guo, Ping Song, Jian Xu, Guang-Rui Wan, Shuang-Xi Wang, Ya-Ling Yin, Peng Li

**Affiliations:** ^1^College of Pharmacy, Henan International Joint Laboratory of Cardiovascular Remodeling and Drug Intervention, Xinxiang Key Laboratory of Vascular Remodeling Intervention and Molecular Targeted Therapy Drug Development, Xinxiang Medical University, Xinxiang 453003, China; ^2^School of Basic Medical Sciences, Xinxiang Medical University, Xinxiang, China; ^3^Department of Ultrasound, First Affiliated Hospital of Xinxiang Medical University, Weihui 453100, China

## Abstract

Diabetic cardiomyopathy (DCM), a cardiovascular complication of patients with diabetes, is a special cardiomyopathy that is independent of coronary heart disease, hypertension, and valvular disease. Citronellal (CT) is a monoterpene compound generated by the secondary metabolism of plants. In this work, the therapeutic effect and mechanism of CT in DCM were investigated. Experimental diabetic rat models were constructed through a high-fat and high-carbohydrate diet combined with low-dosage streptozotocin (STZ) treatment. CT was intragastrically administered at the dosage of 150 mg/kg/day. The cardiac functions of the rats were evaluated via cardiac Doppler ultrasound. Changes in myocardial structure were analyzed through histopathology. Changes in the representative indices of oxidative stress, namely, superoxide dismutase (SOD) activity and malondialdehyde (MDA) content were detected on the basis of a biochemical test. Related protein levels were assayed via immunofluorescence and Western blot analyses. The DCM rats in the nontreatment group experienced diastolic and systolic dysfunctions, associated with myocardial hypertrophy, fibrosis, and cardiomyocyte apoptosis. Moreover, this condition was concurrent with metabolic disorders, the degradation of SOD activity in myocardial tissues, the increase in MDA content, the abnormal activation of sodium–hydrogen exchanger 1 (NHE1), and the aggravation of cell apoptosis (Bax levels were elevated, whereas Bcl-2 levels decreased). Myocardial hypertrophy, fibrosis, oxidative stress, and cell apoptosis were obviously inhibited after treatment with CT (150 mg/kg/day). The abnormal activation of NHE1 was recovered under the action of CT. Our study results showed that CT might play a protective role in the treatment of DCM by repressing the abnormal activation of NHE1.

## 1. Introduction

Epidemiological studies show that three quarters of deaths among patients with diabetes mellitus (DM) are caused by cardiovascular diseases (CVDs) [1, 2]. Diabetic myocardiopathy (DCM), one of the most common and serious complications of patients with DM, is characterized by the abnormality of myocardial structure and functions. However, it is unrelated to coronary heart disease, hypertension, or valvular diseases. The clinical manifestations of DCM are early stage diastolic dysfunction, followed by systolic dysfunction and finally cardiac failure [3]. The cardiac injury mechanism of DM is complicated and triggered by various factors, among which hyperglycemia, oxidative stress, inflammation, myocardial fibrosis, and cell apoptosis may participate in its physiological and pathological processes [4, 5]. Except for blood glucose control, no specific drugs or therapeutic strategy can slow down the progression of DCM [6, 7]. Therefore, deeply probing the pathogenesis of DCM and seeking effective therapeutic methods are of great theoretical and practical importance. DCM can be treated through supplementation with endogenous antioxidants [8]. Hence, chemical compounds with hypoglycaemic and antioxidant characteristics are speculated to contribute to DCM treatment.

*Cymbopogon citratus* Stapf (DC), commonly known as lemon grass, is a perennial herb that is extensively distributed in tropical and subtropical areas. It has been applied in all kinds of foods, chemical engineering, and traditional medical treatment for a long time [9]. In recent years, scientific assessment has revealed that DC is effective in treating CVDs [10]. Essential oil, the main bioactive component of DC, is a very complicated natural mixture that forms after the secondary metabolism of plants. DC essential oil contains substances, such as flavonoids, phenols, and terpenoids, and its main components include citronellal (CT), citronellol, myrcene, and geraniol, with CT content being the highest. CT, a monoterpenoid, is usually separated into the nonracemic mixture of its R and S enantiomers [9, 11, 12]. The already reported pharmacological actions of essential oil include antibacterial [13], anticonvulsant [12], antihypertension [14], and glucose-lowering [15] activities. Moreover, CT has been used as a natural drug in DM treatment in South Africa, Nigeria, and Morocco for a long time [16–18]. Our previous studies found that CT also has a prominent antioxidative stress effect [19].

On the basis of the results of the above studies, citronella essential oil was selected as the drug for the prevention and treatment of DCM. This experiment aims to evaluate the therapeutic effect of CT on DCM, discuss its therapeutic mechanism, and further facilitate the research and comprehensive development of the pharmaceutical values of citronella and promote the discovery of safe and effective natural drugs for the prevention and treatment of DCM.

## 2. Materials and Methods

### 2.1. Materials

CT was provided by Sigma with a purity of ≥95.0%. Streptozotocin (STZ) was purchased from Sigma (Sigma Aldrich, St. Louis, MO, USA) and used to induce experimental diabetes. Primary antibodies against sodium–hydrogen exchanger 1 (NHE1), Bax, and Bcl-2 were obtained from Affinity (Affinity Biosciences, OH, USA). Superoxide dismutase (SOD) and malondialdehyde (MDA) detection kits were provided by Jiancheng Bioengineering Institute (Nanjing, China).

### 2.2. Animals

Male SD rats (180–220 g) were purchased from Henan Experimental Animal Centre and reared in the SPF-level animal house of Xinxiang Medical University at the temperature of 21°C ± 1°C and humidity of 40%–60%. The light/dark period was 12 h, and the rats were fed with pure water and conventional feed. The animal experimental scheme passed the review of the Animal Nursing and Use Commission of Xinxiang Medical University. All experiments were carried out in strict accordance with the suggestions specified in The Guide for Care and Use of Laboratory Animals of the National Institutes of Health.

### 2.3. Modelling and Experimental Grouping

After adaptive feeding for 1 week, the rats were randomly divided into the control group and experimental group. The control group was kept on a normal diet (crude protein 180 g/kg, crude fat 40 g/kg, crude fiber 50 g/kg, crude ash 80 g/kg, calcium10∼18 g/kg, total phosphorus 6–12 g/kg, lysine8.2 g/kg, and methionine and cystine5.3 g/kg) during the experimental process. In reference to related literature [20], type-2 diabetes models were established with the rats in the experimental group as follows: the rats were given a high-carbohydrate diet (diet included 81.5% basic diets, 10% lard, 0.5% sodium cholate, 3% cholesterol, and 5% sugar) throughout the whole experimental period. Four weeks later, the diabetes models were constructed through the intraperitoneal injection of STZ (40 mg/kg) for 5 consecutive days. After 7 days, blood glucose levels were detected via the caudal veins of the rats. If fasting blood glucose levels satisfied ≥16.7 mmol/L twice consecutively, the rat diabetes model was considered to be successful and included in the follow-up experiment. Cardiac Doppler ultrasound showed left ventricular diastolic hypofunction at 6 weeks after STZ injection, indicating initial progression into DCM. Therefore, the DCM modelling was successful.

The experimental groups were as follows: (1) control group; (2) CT (150 mg/kg/day) group; (3) DCM group; (4) DCM + CT (150 mg/kg/day) group. The corresponding dosage of CT was given to the CT control group and drug treatment group through intragastric administration once per day for 16 consecutive weeks. The same volume of physiological saline was given to the control group and DCM group.

### 2.4. Cardiac Ultrasonic Examination

After mildly anesthetizing the animals with pentobarbital, the same doctor evaluated cardiac function and structural abnormality by using a VINNO70 color Doppler ultrasonic cardiogram system. Then, transthoracic 2D m-type ultrasonic cardiograms were acquired. The exported ultrasonic cardiogram parameters included left ventricular end-diastolic volume (LVEDv), left ventricular end-systolic volume (LVESv), fractional shortening rate (FS), ejection fraction (EF), stroke volume (SV), and interventricular septal depth (IVSd).

### 2.5. Assay of SOD Activity and MDA Content in Cardiac Tissues

Cardiac tissue specimens were weighed and SOD activity and MDA content in myocardial tissues were detected via spectrophotometry–colorimetry in accordance with the kits' instruction manuals (Nanjing Jiancheng).

### 2.6. Histopathological Analysis

#### 2.6.1. Determination of Cardiomyocyte Diameter

Specimens were collected from the atrioventricular ring parallel to the heart, fixed with 4% paraformaldehyde, embedded in paraffin, and cut into 5 *μ*m slices. The slices were stained with hematoxylin and eosin (H&E) to observe the change in myocardial structure. Meanwhile, 10 cardiomyocytes were selected from each slice under 400× magnification. The short-axis diameters of the cardiomyocytes were measured, and the mean value was calculated to analyze cardiomyocyte hypertrophy quantitatively.

#### 2.6.2. Glycogen Storage Analysis

The PAS staining method was used to detect the change in the glycogen content of the cardiomyocytes. Five high-power fields (400×) were randomly selected from each specimen. The Image-Pro Plus 6.0 image analysis system was used to determine myocardial glycogen volume fraction (myocardial glycogen volume = field area/total myocardial field area).

#### 2.6.3. Myocardial Fibrosis Analysis

The Masson trichrome staining method and Gomori silver staining method were used to detect the changes in myocardial interstitial and reticular fibers to analyze the cellular and molecular mechanisms of the development of cardiac hypertrophy. The area of myocardial fibrosis was quantified by using a colored high-definition pathological image-text analysis system (HPIAS-1000, Wuhan, China). The myocardial fibrosis degree was the ratio of the fibrosis area to the whole myocardial area.

### 2.7. NHE1 Assay via Immunohistochemistry

Tissue slices and NHE1 (Affinity Biosciences, OH, USA) were incubated overnight at 4°C. The assay was performed by using an IHC detection kit (pv-0023, Beijing Bioss) in accordance with the kit's instruction manual. Positive immunoreaction was visualized by using 3,3′-diaminobenzidine (Beijing Solarbio). Images were captured with a scanning microscope (3DHISTECH, Hungary). NHE1 staining was quantitatively analyzed by using Image-Pro Plus 6 analysis software.

### 2.8. Detection of Bcl-2 and Bax via Immunofluorescence

After hydration, the sections were incubated together with specific primary antibodies overnight at 4°C and then with fluorescent secondary antibodies at 37°C for 1 h. Positive immunoreaction was visualized with fluorescein FITC and CY3 and observed under a fluorescent scanning microscope (3DHISTECH, Hungary).

### 2.9. Western Blot Analysis

Western blot analysis was performed in accordance with the related reference [21]. The protein expression levels of NHE1, Bcl-2, and Bax were detected.

### 2.10. Statistical Analysis

Statistical analysis was performed with SPSS20.0 software. The data are presented as means ± SD. Before this analysis, the data were subjected to the normality test and homogeneity test of variance. Intergroup comparison was carried out by using one-way analysis of variance, and *p* < 0.05 was considered statistically significant.

## 3. Results

### 3.1. CT Relieved STZ-Induced Metabolic Abnormality in DCM Rats

The metabolic characteristics of the experimental animals are shown in Table 1. The weights of the untreated diabetic rats in the DCM group were obviously reduced in comparison with those of the rats in the control group (*p* < 0.05). However, blood glucose and FINs levels in the DCM group were significantly higher than those in the control group (*p* < 0.05). The heart weight/body weight (HW/BW) ratio of the DCM group was higher than that of the control group (*p* < 0.05). In comparison with that of the DCM group, the body weight of the CT treatment group was remarkably increased, whereas the HW/BW ratio was significantly reduced (*p* < 0.05). Moreover, CT could reduce the blood glucose and FINs levels of DCM rats to a great extent (*p* < 0.05).

### 3.2. CT Improved Left Ventricular Dysfunction in DCM Rats

**Figure 1:**
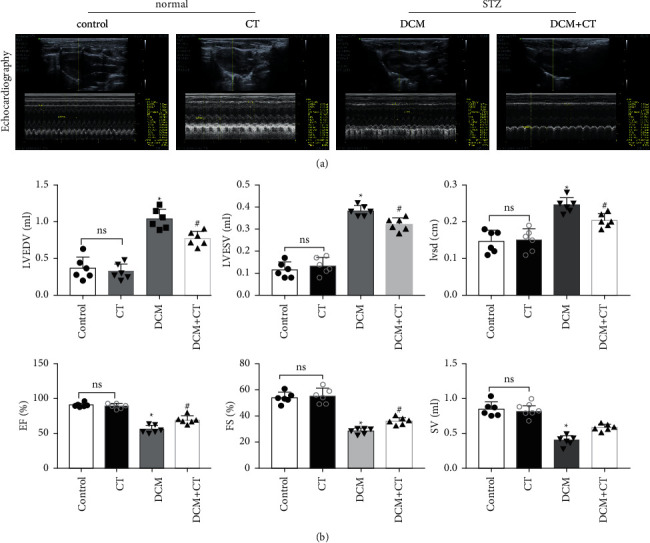
CT improving left ventricular dysfunction in DCM rats. (a) Representative echocardiography of rats in each group. (b) Quantitative analysis of echocardiography parameters. Before the animals were killed, M-mode images were obtained at the papillary muscle level from two-dimensional (2D) short-axis views. LVEDV, left ventricular end-diastolic volume; LVESV, left ventricular end-systolic volume; IVSd, interventricular septal diastolic wall thickness; EF%, ejection fraction; FS%, fraction shortening; SV, stroke volume. All data are expressed as means ± SD; *n* = 6 rats per group; CT, citronellal; DCM, diabetic cardiomyopathy; ^*∗*^*p* < 0.05 vs. control; ^#^*p* < 0.05 vs. DCM; ns, not significant.

Our results suggested that CT improved left ventricular dysfunction in rats with DCM. As shown in Figures 1(a) and 1(b), the cardiac functions of all rats were examined through cardiac ultrasound before they were executed (Figure 1(a)). In comparison with the normal group, the group under treatment with the high-carbohydrate diet combined with the intraperitoneal injection of STZ showed the obvious induction of the formation of rat DCM, elevated levels of LVEDv, LVESv, and IVSd (*p* < 0.05), and reduced EF, FS, and SV (*p* < 0.05). After the intragastric administration of CT, DCM rats showed obviously elevated EF, FS, and SV, but reduced levels of LVEDv, LVESv, and IVSd. However, CT showed no influence on normal SD rats (Figure 1(b)).

### 3.3. CT Preserved Redox State in Rats with DCM

**Figure 2:**
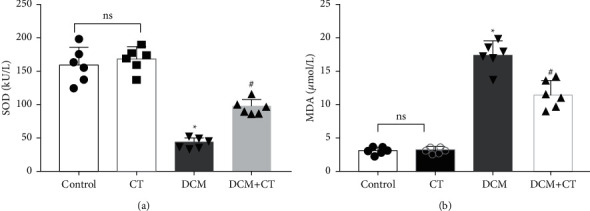
CT preserving redox state in rats with DCM. (a) CT-enhanced SOD activity in heart tissues of diabetic cardiomyopathy rats. SOD, superoxide dismutase. (b) CT-reduced MDA content in heart tissues of diabetic cardiomyopathy rats. MDA, malondialdehyde. Data are mean ± SD; ^*∗*^*p* < 0.05 vs. the control group; ^#^*p* < 0.05 vs. the DCM group; *n* = 6 per group; ns, not significant.

When lipid peroxides were accumulated in the myocardial tissues, SOD activity was reduced, whereas MDA content was increased. SOD activity in the myocardial tissues of the DCM rats was reduced, and MDA content was elevated. After the rats were treated with CT, their SOD (Figure 2(a)) activities and MDA (Figure 2(b)) were recovered.

### 3.4. CT Alleviated Cardiac Pathological Injury in Experimental DCM Rats

**Figure 3:**
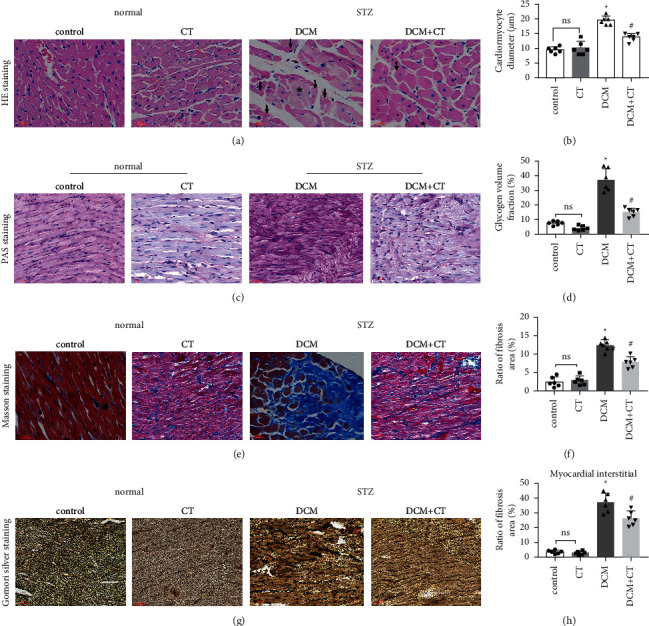
CT alleviating cardiac pathological injury in experimental DCM rats. (a) Representative images of myocardial tissues stained with hematoxylin and eosin (H&E) in each group of rats. Arrow indicates cytoplasmic vacuolization; pentagram indicates cardiomyocyte hypertrophy. (b) Quantitative analysis of cardiomyocyte diameter. (c) Representative images of myocardial tissues stained with periodic acid–Schiff (PAS) in each group of rats. The nuclei are blue and the glycogen is purplish red. (d) Quantitative analysis of glycogen volume fraction. (e) Representative images of myocardial tissues stained with Masson's trichrome in each group of rats. Collagen fibers are blue. (f) Quantitative analysis of collagen fibers area. (g) Representative images of myocardial tissues stained with Gomori silver in each group of rats. (h) Quantitative analysis of reticular fibers area. Magnification = 400×. Data are mean ± SD; *n* = 6 per group; ^*∗*^*p* < 0.05 vs. the control group; ^#^*p* < 0.05 vs. the DCM group; ns, not significant.

The pathological changes in the cardiac tissues of rats in different groups are shown in Figure 3. H&E staining revealed that the cardiomyocytes in the control group were compact, in alignment, and had clear structure. In the DCM model group, the cardiomyocytes were chaotically arranged. Several cardiomyocytes were hypertrophic and distorted, and many cells were vacuolized in the cytoplasm, indicating the fatty degeneration of cardiomyocytes and the enlargement of the intercellular space. Following CT treatment, the above conditions apparently improved (Figure 3(a)). In particular, the evaluation of cardiomyocyte hypertrophy by measuring the size of the left ventricular cardiomyocytes revealed that in comparison with that in the control group, the diameter of cardiomyocytes in the DCM group had significantly enlarged (*p* < 0.05). CT treatment could obviously repress DCM-induced cardiomyocyte hypertrophy (*p* < 0.05) (Figure 3(b)). PAS staining results showed that compared with that in the control group (Figure 3(c)), glycogen storage in the rat myocardial tissues in the DCM group had increased (*p* < 0.05). This effect could be reversed by CT (*p* < 0.05) (Figure 3(d)). Masson trichrome staining and Gomori silver staining revealed that types I and III collagens, respectively, accounted for the maximum quantity of extracellular matrix proteins in the heart (Figures 3(e) and 3(g)). These results showed that a large quantity of collagens had accumulated in the hearts of the DCM rats with apparent fibrosis. However, the change in cardiac fibrosis was significantly relieved after the DCM rats were treated with CT (*p* < 0.05) (Figures 3(f) and 3(h)).

### 3.5. CT Repressed the Apoptosis of DCM Rat Cardiomyocytes by Influencing the Expression of NHE1 Protein

**Figure 4:**
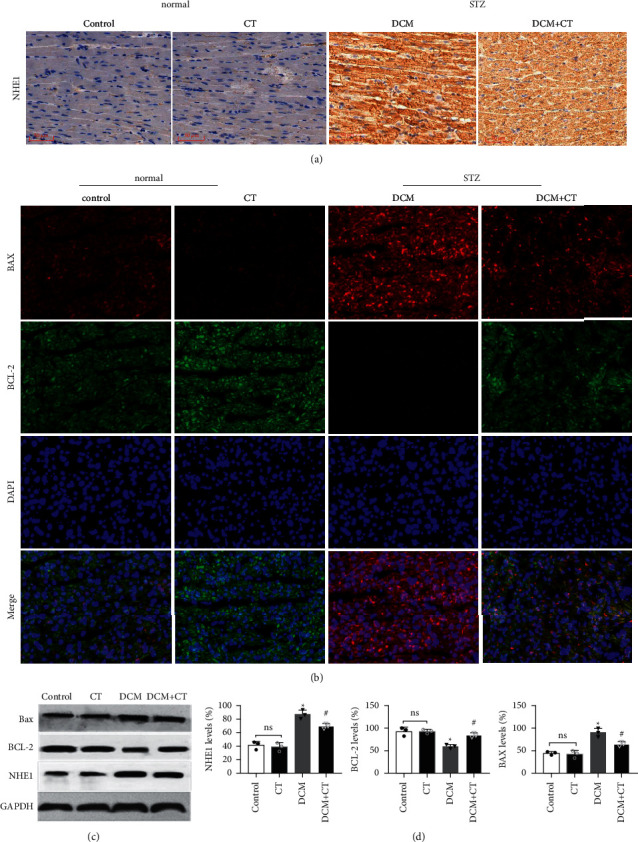
CT repressing the apoptosis of DCM rat cardiomyocytes by influencing the expression of NHE1 protein. (a) Representative immunohistochemical staining of NHE1 expression in each group. (b) Representative immunofluorescence staining of Bcl-2 and Bax expression, respectively. (c) Western blot analysis of NHE1, Bcl-2, and Bax expression, respectively. (d) The quantitative analysis of the expression of NHE1, Bcl-2, and Bax, respectively. *n* = 6 per group. Data are mean ± SD; ^∗^*p* < 0.05 vs. the control group; ^#^*p* < 0.05 vs. the DCM group; ns, not significant.

The abnormal activation of NHE1 protein facilitates cell apoptosis, and myocardial cell apoptosis is considered as an important factor promoting the genesis and development of DCM. Given this situation, the expression levels of NHE1, the antiapoptotic protein Bcl-2, and the proapoptotic protein Bax in myocardial tissues were detected through the IHC technique and Western blot analysis. As shown in Figure 4, when the expression level of NHE1 in the myocardial tissues of DCM rats was elevated, the level of Bax was increased, whereas that of Bcl-2 level was reduced. When DCM rats were treated with CT, the expression level of NHE1 was reversed, the level of Bax declined, and that of Bcl-2 level was elevated (Figures 4(a) and 4(b)), all of which were further proven by Western blot analysis (Figures 4(c) and 4(d)). Therefore, CT might regulate the expression levels of Bax and Bcl-2 by inhibiting the expression of NHE1, thus repressing cardiomyocyte apoptosis.

## 4. Discussion

Citronella is a nontoxic and nonmutagenic high-safety medicinal plant when used at conventional dosage, which has a potential therapeutic effect on a variety of diseases [22, 23]. Its main active components are essential oils. In addition to being familiar spices, these essential oils have a potential therapeutic effect in a variety of diseases. In Nigeria, citronella leaves are used to prepare tea for the adjuvant treatment of hypertension and diabetes [24]. Recent studies have shown that CT can effectively prevent and treat CVDs [25]. In this study, the new application of citronella, an aromatic plant, was extended. This work showed that CT could significantly decelerate the development of rat DCM caused by combined treatment with a high-fat and high-carbohydrate diet and STZ injection and effectively improved DCM-triggered cardiac structural obstruction and dysfunction. Its mechanism might be associated with the reduction in blood glucose levels and the inhibition of oxidative stress and cardiomyocyte apoptosis.

In patients with diabetes, DCM is a common serious cardiovascular complication, the primary factor causing heart failure and the main reason for deaths in advanced stages. Its pathogenesis mechanism may be related to insulin resistance, hyperglycaemic injury, oxidative stress, and cardiomyocyte apoptosis. In this work, the DCM model was established through treatment with a high-fat and high-carbohydrate diet combined with intraperitoneal STZ injection. In this approach, the high-fat and high-carbohydrate diet would first induce insulin resistance. Subsequently, the injection of low-dosage STZ resulted in islet cell dysfunction and insulin secretion reduction. Reliable type-2 diabetes animal models were thus obtained. Consistent with those in previous reports [26], the untreated DCM rats in this experiment exhibited weight loss, polydipsia, polyphagia, polyuria, elevated blood glucose levels, and increased HW/BW ratios. Moreover, the ultrasonic cardiograms showed that DCM rats in the untreated group experienced diastolic and systolic dysfunctions, which correlated with myocardial hypertrophy and collagen accumulation in the myocardial interstitium.

Previous studies have shown that the occurrence of cardiovascular complications in patients with DM can be postponed by reductions in blood fat and blood glucose levels [27, 28]. Adeneye administered *C. citratus* aqueous extract (125–500 mg/kg) to normal Wistar rats orally once per day. After 42 days, the rats presented dosage-dependent weight loss and reduced blood glucose and blood fat [29]. Our previous experiment [19] and this experiment also verified that citronella essential oil indeed had the effects of lowering blood fat and blood glucose. In this experiment, the oral administration of CT reversed weight loss and reduced blood glucose level and HW/BW ratio in the experimental diabetes rats.

CT could exert a therapeutic effect on DCM by repressing oxidative stress. SOD activity could be enhanced, and MDA content could be reduced by the oral administration of CT. Previous reports [8] have indicated that when a living organism lives in a hyperglycaemic environment, the in vivo generation of reactive oxygen species (ROS) is increased, the activity of the antioxidant enzyme SOD is degraded, and the capability of the living organism to scavenge ROS is greatly weakened, thus generating oxidative stress that then leads to myocardial lipid peroxidation injury. MDA, a stable metabolite of lipid peroxidation, is regarded as an indirect index of ROS generation, and its content reflects the degree to which the cells in the living body are attacked by free radicals. The injury degree of cardiomyocytes and the protective degree of drug intervention for cardiomyocytes were determined by detecting SOD activity and MDA content in myocardial tissues. The result found in this work coincided with the experimental result obtained by Gayathri et al. [10], who demonstrated that the preventive administration of citronella extract (200 mg/kg) has an obvious protective effect on isoproterenol-induced cardiomyocyte injury in rats. This protective mechanism may be correlated with the scavenging of free radicals, the alleviation of lipid peroxidation, and the enhancement in antioxidant enzyme activity.

How CT decelerates the development of DCM remains a question. As far as we know, this effect may be correlated with the inhibition of the abnormal activation of NHE1 by CT. NHE1 belongs to the NHE family. As a transmembrane protein that extensively exists in cells, NHE1 mediates the exchange of extracellular Na^+^ with intracellular H^+^ at the proportion of 1 : 1 to maintain the intracellular pH value and cell volume under physiological conditions. Moreover, it is the subtype that is mainly expressed in the cardiomyocytes of human beings and mammals [30]. Past studies have shown that NHE1 is closely related to the genesis and development of cardiovascular and cerebrovascular diseases. NHE1 activity in cardiomyocytes is significantly elevated in the hearts of animals and human beings in the final stage of chronic cardiac failure [31, 32]. Jandeleit-Dahm [33] experimented on the mesenteric vessels of rats with STZ-induced diabetes and found that the mesenteric vascular hypertrophy of diabetic rats is associated with the activation of NHE1 and that the NHE1 inhibitor CARIPORIDE can reverse vascular hypertrophy. He then proposed that NHE1 inhibition is a potential target for coping with the rapid development of diabetic vascular diseases. It has been demonstrated that the activity of NHE1 is abnormally elevated at the onset of diabetes. Inhibiting NHE1 improved vascular endothelial functions in diabetes and reversed vascular hypertrophy and myocardial lesions [34, 35]. Our study showed that NHE1 levels in untreated DCM rats were obviously higher than those in the normal group. CT, if taken orally, can repress NHE1 expression, inhibit cardiomyocyte hypertrophy, reduce glycogen storage, and alleviate the fatty degeneration and fibrosis of cardiomyocytes to improve the cardiac functions of DCM rats obviously. However, the concrete action mechanism of this effect remains to be further explored.

The increase in cell apoptosis is the main pathological change in the development of DCM. The loss of cardiomyocytes damages cardiac structure and functions. The levels of Na ions are increased due to overactivation of NHE1. Given the exchange between Na and Ca ions, intracellular Ca ion levels also increase, and Ca overload can activate the mitochondrial permeability transition pore, activate the mitochondrial apoptosis pathway, and lead to cell death [36]. The cell apoptosis-related genes Bcl-2 and Bax were detected in this work. Bcl-2 is a representative mitochondrial inner membrane gene that inhibits cell apoptosis. In contrast to Bcl-2, Bax facilitates cell apoptosis. In our study, compared with those in the control group, Bcl-2 expression levels were significantly lower (*p* < 0.05) and Bax expression levels were significantly higher (*p* < 0.05) in the DCM rats, indicating that cardiomyocyte apoptosis was enhanced during DCM. After CT treatment, Bcl-2 levels were significantly elevated and Bax levels were apparently reduced (*p* 0.05) in the treatment group, indicating that CT could repress cardiomyocyte apoptosis and exert a protective effect on cardiomyocytes. This result was identical to the experimental result obtained by Javadov et al. [30] who applied the NHE1 inhibitor cariporide to the coronary arteries of ligated rats and found that NHE1 can improve mitochondrial functions and reduce cardiomyocyte apoptosis.

## 5. Conclusions

This study provided support for the new function of CT. Specifically, citronella essential oil exerted potential protective and therapeutic effects on DCM. DC, a natural aromatic plant, remitted metabolic disorders, oxidative stress, myocardial fibrosis, and cell apoptosis in DCM rats, thus improving cardiac functions. This cardiac protective effect was associated with the repression of abnormal NHE1 activation.

## Figures and Tables

**Table 1:** CT alleviates metabolism abnormalities.

Group	Bodyweight (g)	HW/BW (mg/g)	Blood glucose (mmol/L)	FINs (*µ*IU/ml)
Control	428 ± 32	2.79 ± 0.22	5.2 ± 0.45	23.83 ± 4.26
CT	389 ± 33	2.78 ± 0.37	5.1 ± 0.57	23.67 ± 2.58
DCM	317 ± 31^*∗*^	3.50 ± 0.41^*∗*^	21.4 ± 1.05^*∗*^	37 ± 4.56^*∗*^
DCM + CT, 150 mg/kg	368 ± 34^#^	3.01 ± 0.21^#^	8.9 ± 0.66^#^	30 ± 3.74^#^

CT, citronellal; DCM, diabetic cardiomyopathy. The rats were measured for their weight and heart weight on the day they were killed. Blood glucose and FINs under basal fasting state was measured on the day the rats were killed. Data are means ± SD; ^*∗*^*p* < 0.05 vs. the control group; ^#^*p* < 0.05 vs. the DCM group; *n* = 6 per group.

## Data Availability

The data used to support the findings of this study are available from the corresponding author upon request.
